# Morpho-histology, endogenous hormone dynamics, and transcriptome profiling in *Dacrydium pectinatum* during female cone development

**DOI:** 10.3389/fpls.2022.954788

**Published:** 2022-08-17

**Authors:** Enbo Wang, Wenju Lu, Haiying Liang, Xumeng Zhang, Shaojie Huo, Xiqiang Song, Jian Wang, Ying Zhao

**Affiliations:** ^1^Key Laboratory of Ministry of Education for Genetics and Germplasm Innovation of Tropical Special Trees and Ornamental Plants, Key Laboratory of Germplasm Resources Biology of Tropical Special Ornamental Plants of Hainan Province, College of Forestry, Hainan University, Haikou, China; ^2^Department of Genetics and Biochemistry, Clemson University, Clemson, SC, United States

**Keywords:** conifer, gene expression, endogenous hormone, phenology, RNA sequencing, reproductive development

## Abstract

*Dacrydium pectinatum* de Laubenfels is a perennial dioeciously gymnosperm species dominant in tropical montane rain forests. Due to deforestation, natural disasters, long infancy, and poor natural regeneration ability, the population of this species has been significantly reduced and listed as an endangered protected plant. To better understand the female cone development in *D. pectinatum*, we examined the morphological and anatomical changes, analyzed the endogenous hormone dynamics, and profiled gene expression. The female reproductive structures were first observed in January. The morpho-histological observations suggest that the development of the *D. pectinatum* megaspore can be largely divided into six stages: early flower bud differentiation, bract primordium differentiation, ovule primordium differentiation, dormancy, ovule maturity, and seed maturity. The levels of gibberellins (GA), auxin (IAA), abscisic acid (ABA), and cytokinin (CTK) fluctuate during the process of female cone development. The female cones of *D. pectinatum* need to maintain a low level of GA_3_-IAA-ABA steady state to promote seed germination. The first transcriptome database for female *D. pectinatum* was generated, revealing 310,621 unigenes. Differential expression analyses revealed several floral (*MADS2*, *AGL62*, and *LFY*) and hormone biosynthesis and signal transduction (*CKX*, *KO*, *KAO*, *ABA4*, *ACO*, etc.) genes that could be critical for female cone development. Our study provides new insights into the cone development in *D. pectinatum* and the foundation for female cone induction with hormones.

## Introduction

*Dacrydium pectinatum* de Laubenfels is a perennial dioeciously gymnosperm of *Dacrydium* in Podocarpaceae (De and David, 1969). *D. pectinatum* is mainly distributed in Brunei Darussalam, China (Hainan), Indonesia (Kalimantan, Sumatera), Malaysia (Sarawak, Sabah), and the Philippines. *D. pectinatum* is the only representative species of this genus distributed in China, and it is the key species to building the natural community of mountain rain forests (Huang et al., 2014). Therefore, the study of *D. pectinatum* is of scientific significance to reveal the origin and flora of tropical forests. *D. pectinatum* can live for more than 2,000 years and grow up to 30 m tall and 3 m in diameter at breast height. The wood of *D. pectinatum* is precious and of high quality, with excellent texture and hardness, and specific gravity 0.60∼0.71. It can be used in architecture, shipbuilding, furniture, etc. The oil content of the *D. pectinatum* seeds is 17.5%, and the bark contains ecdysone and biflavone, which show good activity in anti-tumor, anti-virus, anti-oxidation, and anti-inflammatory effects (Coulerie et al., 2013; Bagla et al., 2014). However, due to excessive logging and shifting cultivation, there is a significant decrease in the species’ natural forest resources, and it is now listed as Endangered under criteria A4acd (IUCN 3.1) by the International Union for Conservation of Nature. Massive conversion of lowland forest (kerangas) on the coastal plains of Sabah, Sarawak, and Kalimantan to oil palm plantations has reduced *D. pectinatum* probably to 20% of its former area of occupancy.^1^ Similarly, *D. pectinatum* natural forests have been significantly reduced in China since the 1960s due to severe damage by excessive deforestation, typhoons, and other external forces. According to a report published in 2016 (China Forestry Bureau), only 14,484 hectares are available in China, accounting for 3,473,575 cubic meters of forest volume.

A survey of *D. pectinatum* found that 78% of trees in natural forests had a large diameter at breast height (> 5cm) and natural regeneration was poor in the montane rain forests (Ding et al., 2012; Wu et al., 2018). Currently, the approximate length of the juvenile phase is 20 years for *D. pectinatum* since none of the trees with a DBH of 10 cm or smaller produced reproductive structures. Among the 180,032 *D. pectinatum* seeds collected from a natural stand in the Hainan province, China, the viability and germination rate were found to be 3.11 and 0.02%, respectively (Chen et al., 2016). Moreover, the number of female trees is less than that of the male trees, and the flowering rate is low, which seriously affects the pollination ratio, and then affects the regeneration of *D. pectinatum*. At present, multi-omics technology has greatly promoted people’s understanding of plant development mechanisms. The huge genome of gymnosperms limits the pace of research on such plants. Recently, high-quality genome assembly and annotation have been completed in *Pinus tabuliformis* and *Cycas panzhihuaensis* (Liu et al., 2022; Niu et al., 2022). In *D. pectinatum*, only male transcriptome data were used to study the development of male cones (Lu et al., 2021). Information about the floral regularity of *D. pectinatum* female cones is still blank. These factors have seriously hindered the artificial cultivation of *D. pectinatum* and the protection and effective utilization of resources.

Efforts have been undertaken to protect *D. pectinatum* and conserve its biodiversity. However, research on this endangered species is still in its infancy. Currently, most of the published studies are focused on the activity of medicinal ingredients, seedling growth, forest community structure, genetic diversity, and origin of evolution (Liang and Yin, 1994; Su et al., 2010; Keppel et al., 2011; Huang et al., 2014; Wu et al., 2018; Yang et al., 2018). Limited information is available on the reproduction of the species in *Dacrydium*. For example, the developmental regularity of female cones and what factors contribute to the low seed quality and poor natural regeneration is unknown. This study aimed to reveal the developmental mechanism of the male and female cones of *D. pectinatum* and help fill the vast knowledge void urgently needed for the conservation and propagation of the endangered species. We employed a combined approach of anatomy, hormone dynamics, and gene expression to study the development process of *D. pectinatum* female reproductive structure. Hormones play critical roles in development, including reproduction. For instance, applying hormones has become an effective practice to induce reproductive bud initiation, especially in species with long juvenility. Liang and Yin (1994) successfully reduced cone production age from 25 to 5 years with indole butyric acid (IBA) and zeatin ribonucleoside (ZR) in *Metasequoia glyptostroboides* (Liang and Yin, 1994). Male strobili of *Cryptomeria japonica* were induced by GA_3_ spraying onto the shoots (Huang et al., 2014). To be effective, exogenous phytohormones need to be applied before and/or during early bud differentiation. Understanding the molecular mechanism of *D. pectinatum* cone development shall pinpoint critical genes that can be utilized in early cone induction. Because no prior large-scale *Dacrydium* genomic resources were available and RNA sequencing is a common approach to the discovery of target genes, we subjected female cones at six timepoints to RNA sequencing, generating the first transcriptome dataset for female *D. pectinatum* and revealing the genes important in female cone development. The new insights from our study lay a foundation for solving the problems of low germination rate and long juvenile phase of *D. pectinatum* seeds.

## Materials and methods

### Plant material and growth conditions

Female reproductive structures of *D. pectinatum* were collected monthly from early January 2019 to December 2020 at the Bawangling Forest Reserve (between 18°53′ and 19°30′ north latitude, between 108°38′ and 109°17′ east longitude) of Changjiang County, Hainan Province, China. The collection of samples was approved by the Hainan Bawangling Nature Reserve Department, which complies with national and international standards. The species were previously confirmed by botanists and labels were present on the trees. Our conduct complied with the Convention on the Trade in Endangered Species of Wild Fauna and Flora. Changjiang County is in a typical tropical monsoon climate zone. The annual average temperature is 24.3°C, with 39.8°C as the highest and 0°C as the lowest. There is no winter throughout the year. The annual accumulated temperature is 8,400∼9,100°C, while the total solar radiation is 135 kcal/cm^2^. Rainfall is abundant with an average annual precipitation of 1,676 mm. Female buds were collected from the north, south, east, and west sides of four mature trees that are over 100 years old based on the available records. A total of ten cones from each collection date were used for size measurement. Bracts were dissected and removed under a SMZ-168 stereomicroscope before buds were stored at 4°C in 2.5% glutaraldehyde or a fixative solution (formalin: acetic acid: 70% alcohol = 1:1:18, FAA). Photos were taken of female shoots at different stages of development with a Nikon digital camera.

### Semi-thin sectioning

Female buds fixed in FAA were dehydrated and embedded in paraffin as described in Kurita et al. (2020). Sections (∼4°μm) were cut with a microtome (RM2016, Shanghai, China) and mounted on slides. After rehydration, specimens were stained with 1% saffron and 0.5% solid green and observed under a Nikon Eclipse Ci light microscope (Tokyo, Japan). Digital images were taken with a Nikon digital camera.

### Observation of female cone morphology by scanning electron microscopy

Female buds fixed in 2.5% glutaraldehyde were treated with 1% osmium acid. After dehydration with a series of increasing concentrations of ethanol and dipping into isoamyl acetate, the dried samples were sputter-coated with gold prior to scanning electron microscopy examination (SEM, U8010, HITACHI, Tokyo, Japan) according to Kang et al. (2014).

### Detection of endogenous hormones

Female reproductive samples were collected at various timepoints from the four mature trees mentioned above from January 2019 to December 2020 and preserved at −80°C. The frozen samples were ground into fine powder with a grinding machine (30 Hz, 1 min). After ground samples of the same timepoint were equally pooled 50 mg were weighed and dissolved in a 0.5 ml-extract solution containing methanol, water, and formic acid (v:v:v = 15:4:1). After 10 min of extraction, the supernatant was obtained by centrifugation for 5 min at 14,000 rpm. The extraction and centrifugation steps were repeated twice. All supernatants were combined and dried at 35°C under nitrogen gas. The extracts were then resuspended with 100 μl of an 80% methanol–water solution and sonicated for 1 min, followed by filtration through a 0.22-micron polytetrafluoroethylene membrane.

Auxin (IAA), cytokinin (CTK), abscisic acid (ABA), and Gibberellic acid 3 (GA_3_) were detected by enzyme-linked immunosorbent assay (ELISA), following the manufacturer’s instructions (Jingmei Biotechnology, China). The detailed procedures are described in Ma et al. (2008). The hormonal contents obtained in the analysis were expressed as pg or μg per gram fresh weight. For each timepoint, three samples were separately prepared and analyzed.

### Ribonucleic acid sequencing and *de novo* transcriptome assembly

Female reproductive tissues representing the six developmental stages, ovuliferous scale development and formation stage (January 12), ovule formation stage (February 18), pollination period (March 14), fertilized ovule development stage (May 11), fertilized ovule development stage (July 15), seed maturity (October 20) as well as vegetative tissues (leaves) at the same timepoints were harvested from three of the above-mentioned mature trees, quickly frozen in liquid nitrogen, and then sent to the facility in Novogene (Beijing, China) for RNA extraction, cDNA library construction, and paired-end sequencing (Illumina HiSequation 4000). There were three biological replicates per timepoint and tissue type (female buds or leaves). Therefore, there were a total of 36 samples for RNA sequencing. Detailed protocols for RNA-Seq and analyses can be found in He et al. (2018). Briefly, RN38 EASYspin Plus Plant RNA Kits (Aidlab Biotechnologies Co., Ltd., Beijing, China) were used for RNA extraction, RNase-free DNase I was applied to remove residual DNA. The RNA integrity of these samples was assessed using the RNA Nano 6000 Assay Kit of the Agilent Bioanalyzer 2100 system (Agilent Technologies, Santa Clara, CA, United States). mRNA-Seq libraries were constructed using the NEBNextUltra RNA Library Prep Kit for Illumina (New England Biolabs, CA, United States).

After removal of adaptor sequences and low-quality bases at the 3′ end, as well as reads with >10% unknown nucleotides from the 5′ and 3′ ends of the remaining reads or bases of Q-score ≤10% being more than 50%, the high-quality clean data were used to perform *de novo* assembly. Transcriptome assembly was performed by assembling clean reads into contigs, transcripts, and unigenes using Trinity software with default values^2^ (Grabherr et al., 2013; Hu et al., 2014). The resulting contigs were clustered into unigenes by Corset (Davidson and Oshlack, 2014) before being compared against the following databases: National Center for Biotechnology Information (NCBI) non-redundant protein sequences (NR), NCBI non-redundant nucleotide sequences (NT), a manually annotated and reviewed protein sequence database (Swiss-Prot), Gene ontology (GO), euKaryotic Orthologous Groups (KOG), Kyoto encyclopedia of genes and genomes (KEGG), and Protein family (Pfam), with an *E*-value ≤ 10^–5^ for the functional annotation.

### Differentially expressed gene selection

Expression levels were calculated as fragments per kilobase of transcript per million mapped reads (FPKM) for each sample. DEGs were identified in the female reproductive tissues at six different timepoints as well as in comparison to vegetative tissues. The DESeq R package (1.10.1) was employed. Based on the negative binomial distribution model, the DESeq software estimates variance-mean dependence in count data from high-throughput sequencing and tests for differential expression (Anders and Huber, 2010). The resulting *p*-values were adjusted using Benjamini and Hochberg’s approach for controlling the false discovery rate. Genes with an adjusted *p*-value < 0.05 and Fold Change (FC) ≥ 1 found by DESeq were assigned as differentially expressed.

To verify the reliability of the RNA sequencing data, reverse transcription-quantitative polymerase chain reactions (RT-qPCR) were conducted with a set of eleven DEGs related to the endogenous hormone synthesis pathway and using the female cones in five different periods’ collections from RNA sequencing. Total RNA was extracted using a Qiagen RNeasy Mini Kit (Qiagen Inc., Valencia, CA, United States) and was reverse transcribed into cDNA by using random primers with HiScript III RT SuperMix for qPCR (+ gDNA wiper) (Vazyme, Nanjing, China). The internal control was a translation elongation factor (EF1-α). The 2 × TsingKe^®^ Master qPCR Mix-SYBR (+ UDG) (Tsingke, Beijing, China) was used for RT-qPCR. The 2^–ΔΔ^C*^T^* method was used to analyze the data (Livak and Schmittgen, 2001). The sequences of primers are listed in Supplementary Table 1.

### Statistical analysis

Unless otherwise indicated, Student’s *t*-test and Fisher’s Least Significant Difference (LSD) multiple comparisons were used for statistical analyses at the confidence level of 95%. IMB^®^ SPSS version 22 was utilized.

## Results

### Arrangement and phenology of female cones

**FIGURE 1:**
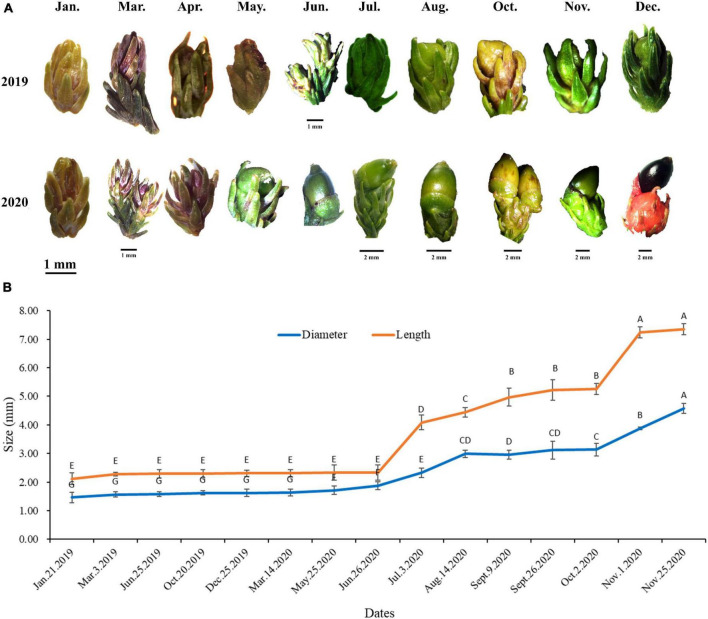
External morphological images **(A)** and size **(B)** of female cones of *Dacrydium pectinatum* at different developmental stages. Specimens were collected from Changjiang County, Hainan Province, China. Different letters among length or diameter indicate significant difference at *p* < 0.05, and *N* = 10.

**FIGURE 2:**
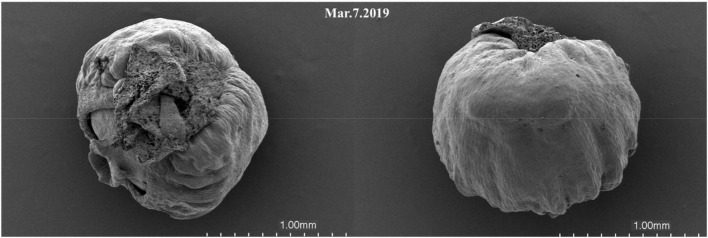
Scanning electron microscope images of female cones of *Dacrydium pectinatum* at ovule formation stage.

Female cones grow alone on the top of branches, sessile. The seed of *D. pectinatum* is ovoid, about 7 mm long, 4 mm in diameter, blunt at the apex, transverse in the thin and dry cup-shaped pseudotesta, red or maroon at maturity (Figure 1). In Bawangling Forest Reserve, female reproductive structures were first observed in January, with a straw yellow color and a diameter of 1.46 mm, and a length of 2.12 mm. The female cone elongated and expanded slowly in the first year. By December, the diameter reached 1.62 mm, the length was 2.31 mm, and the green color deepened (Figure 1). From March to April of the following year, the female strobilus completed fertilization. During seed maturation, the female cones expanded rapidly from June to December. By late November, the average length of cones reached 7.35 mm and the diameter reached 4.57 mm (Figure 1B). By December, the cones gradually turned dark green to maroon, and the seeds matured (Figure 1A). Observation of the female cone development was carried out from 2019 to 2020. The SEM images show that the ovule has developed and was formed in March (Figure 2).

### Microscopic anatomy of *Dacrydium pectinatum* female cones

**FIGURE 3:**
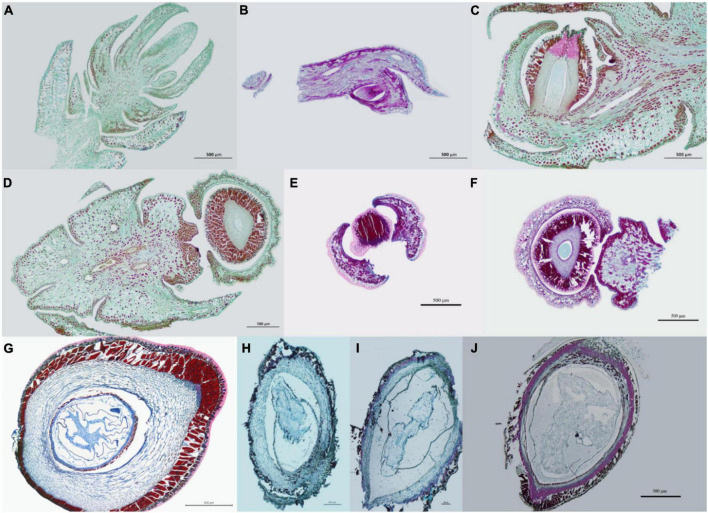
Microscopic images of female cones of *Dacrydium pectinatum* at different developmental stages. **(A,B)** Flower bud differentiation stage. (**A**: January.25.2019; **B**: February.25.2019); **(C–E)** Bract primordium differentiation stage. (**C**: March.25.2019; **D**: April.25.2019; **E**: May.10.2019); **(F–H)** Ovule development and maturation stage. (**F**: June.25.2019; **G**: July.25.2020; **H**: August.25.2020); **(I,J)** Seed maturation stage. (**I**: September.25.2020; **J**: October.25.2020).

In January and February of the first year, flower bud primordium started to become visible, which formed on the top of the bud (Figures 3A,B). In March, the bract primordium began to differentiate and continued until May (Figures 3C–E). The ovules began to develop in June and matured from August of the second year (Figures 3F–J). During ovule development, the female cones experience a dormancy period of about 3 months from the winter of the first year to the next year.

After the pollination of female cones of *D. pectinatum* in the first year, the slightly open ovuliferous scales are close to each other and “closed.” Pollen grains germinate soon after entering the micropyle and grow pollen tubes. After the pollen tubes penetrate the nucellus for a certain distance, they stop growing. At the same time, the megaspore mother cell meiosis divides into four megaspores, three degenerate, one continues to develop, divides and produces 16 to 32 free nuclei, and then enters the dormant state in December. From February of the second year, it continues to divide to produce thousands of free nuclei, and then produces the cell wall to form the female gametophyte composed of thousands of cells. Several cells near the micropylar end become the primitive cells of the archegonial initial, which divide and develop into several archegonial initial, including egg cells. The corresponding pollen tube continues to elongate, reaches near the egg cell, releases sperm, and the sperm fuses with the egg cell to complete fertilization. Fertilization is completed in about 13 months after pollination. Therefore, the cones of *D. pectinatum* generally mature in 2 years. After female flowers pollinate in the first spring, they bloom in the second spring and mature in autumn.

### Dynamics of endogenous hormones during female cone development

**FIGURE 4:**
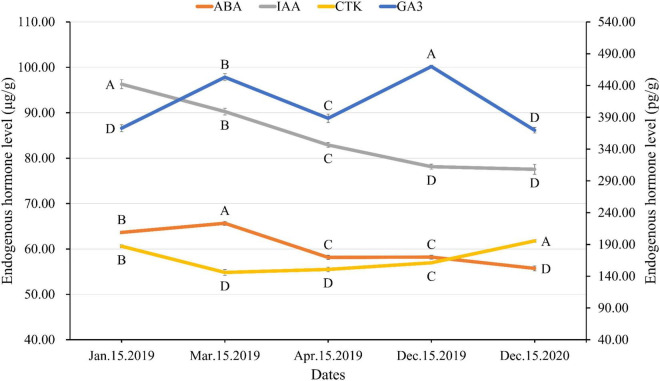
Endogenous hormone changes in female cones of *Dacrydium pectinatum* at different developmental stages. Hormones analyzed by ELISA. The unit of GA_3_ was pg/g fresh weight (right vertical axis). The unit for abscisic acid, auxin, and cytokinin was μg/g fresh weight (left vertical axis). Different letters indicate significant difference at *p* < 0.05, *N* = 3.

As female cones developed, the GA_3_ level gradually increased from January to March, began to decline from March to April, and finally increased to its peak in December. By December of the following year, the GA_3_ level decreased to the lowest level (Figure 4A). The IAA level peaked in January, corresponding to the stage of ovuliferous scale development and ovule formation stage. After that, the IAA level gradually decreased with the development process (Figure 4A). CTK showed the lowest level in March. Because the development of female cones in January is in the stage of ovuliferous scale and ovule formation, and the stage of seed maturation after pollination in March involves active cell division, it is reasonable to have a high level of CTK in these stages. ABA showed a high level in January and March of bead scale development and ovule formation. Subsequently, the ABA level gradually decreased to the lowest in December of the second year.

### *De novo* assembly of *Dacrydium* transcriptome

The sequencing of 36 samples representing leaf and female cone at six timepoints generated a total of 6.87 billion base pairs with an average of 22,425,437 raw sequencing reads and 22,226,803 clean reads per sample (Supplementary Table 2). These clean reads are available in the NCBI Short Read Archive (accession number: PRJNA842859). The average ratio of clean reads to raw reads was 99.97%, and the average Q20 rate was 97.84%. The correlation between replication did not reach 0.8 only in the Mtr5, Mtr7, and Mtr10 groups (Supplementary Figure 1). Moreover, in the principal component analysis of *D. pectinatum* RNA-Seq samples, the three biological repeat samples of F7 were not clustered together (Supplementary Figure 2). So, we removed the data of F7 in the subsequent gene differential expression analysis. In total, 310,621 unigenes were generated. The proportion of unigenes in each sample type ranged from 79.04 to 83.64% (Supplementary Figure 3). The average unigene length is 861 bp, with 14,202 unigenes > 2,000 bp, 26,329 between 1,001 and 2,000 bp, 60,894 between 500 and 1,000 bp, and 82,668 ≤ 500 bp. The overall GC content was 44.99%.

**FIGURE 5:**
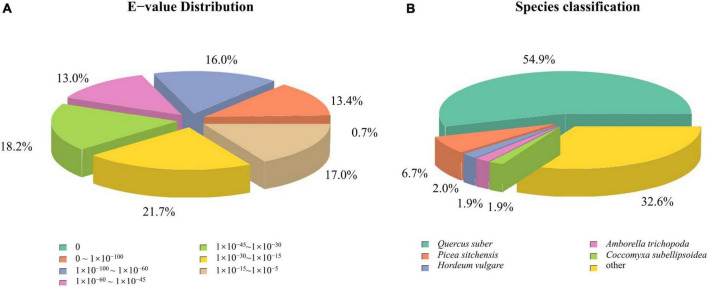
Distribution of *E*-values **(A)** and species classification **(B)** of *Dacrydium pectinatum* unigenes when aligned in the National Center for Biotechnology Information (NCBI) non-redundant protein sequences.

When compared to the seven common public datasets (NR, NT, KEGG, Swiss-Prot, Pfam, GO, and KOG), 70.42% of the unigenes were annotated in at least one of the datasets, with 58.81% in NR, 49.18% each in Pfam and GO, 51.16% in Swiss-Prot, 26.05% in NT, 26.78% in KEGG, and 27.5% in KOG. A set of 18,724 unigenes (10.17%) found annotations in all seven datasets. Among the matches within NR, 13.4% unigenes had an *E*-value < 10^–100^, while the *E*-value range of 10^–100^ to 10^–60^ accounted for 16% of the unigenes (Figure 5A). Among the plant species surveyed in Figure 5B, the *Dacrydium* unigenes had the most matches (54.9%) with cork oak (*Quercus suber*), followed by *Picea sitchensis* (6.7%), a coniferous tree. When the annotated functions were classified with KOG, the top four were translation, ribosomal structure and biogenesis (8,661), posttranslational modification, protein turnover, and chaperones (7,993), general function prediction only (5,390), and energy production and conversion (5,167) (Supplementary Figure 4). Nuclear structure, extracellular structures, and cell mobility had the lowest unigenes, 186, 70, and 25, respectively. When annotated with KEGG, the top three pathways were ribosome (5.64%), carbon metabolism (3.24%), and protein processing in the endoplasmic reticulum (2.49%) (Supplementary Table 3). In the GO annotations, the top three categories in biological process were cellular process, metabolic process, and biological regulation. Cellular anatomical entity, intracellular, and protein-containing complex were the top three categories in cellular component, and binding and catalytic activities were the top two in molecular function (Supplementary Figure 5).

### Differentially expressed *Dacrydium* unigenes

**FIGURE 6:**
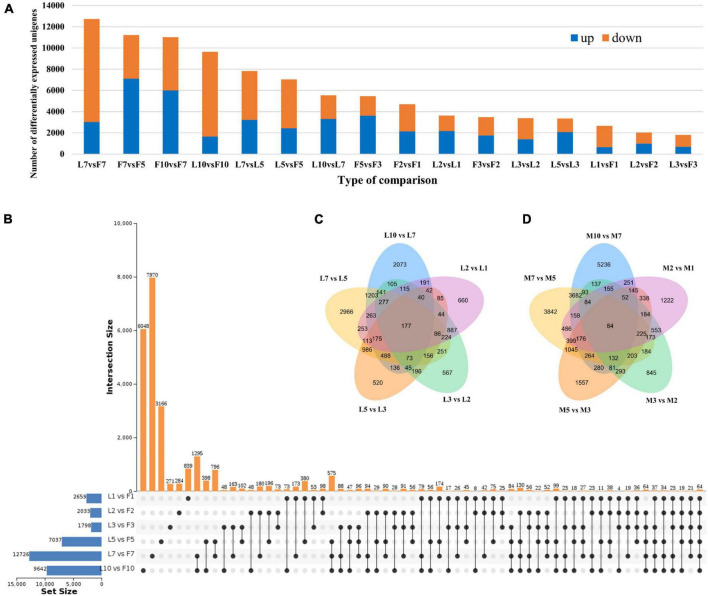
**(A)** Distribution of differentially expressed *Dacrydium pectinatum* unigenes identified in various comparisons. Venn diagram of differential unigenes in L vs. F **(B)**, L vs. L **(C)**, M vs. M **(D)**. L, leaves; F, female cones. 1: January, 2: February, 3: March, 5: May, 7: July, 10: October.

Comparisons among different timepoints and between male reproductive tissue and leaf identified a total of 95,510 DE unigenes, with 42,161 being upregulated and 53,349 being downregulated. As shown in Figure 6A, the comparisons of L7 vs. F7 identified the most DE unigenes, 12,726. In comparison, L3 vs. F3 generated the fewest unigenes, 1,798. A set of unique DE unigenes, 7,970; 6,048; 3,166; 839; 284; and 271, were found in L7 vs. F7, L10 vs. F10, L5 vs. F5, L1 vs. F1, L2 vs. F2, and L3 vs. F3, respectively, when comparisons were performed between female reproductive tissue and leaf at the same timepoints (Figure 6B). The number of timepoint-specific DE unigenes in the leaf number ranged from 520 (L5 vs. L3) to 2,966 (L7 vs. L5), while in the female cone ranged from 845 (F3 vs. F2) to 5,236 (F10 vs. F7) (Figures 6C,D).

**FIGURE 7:**
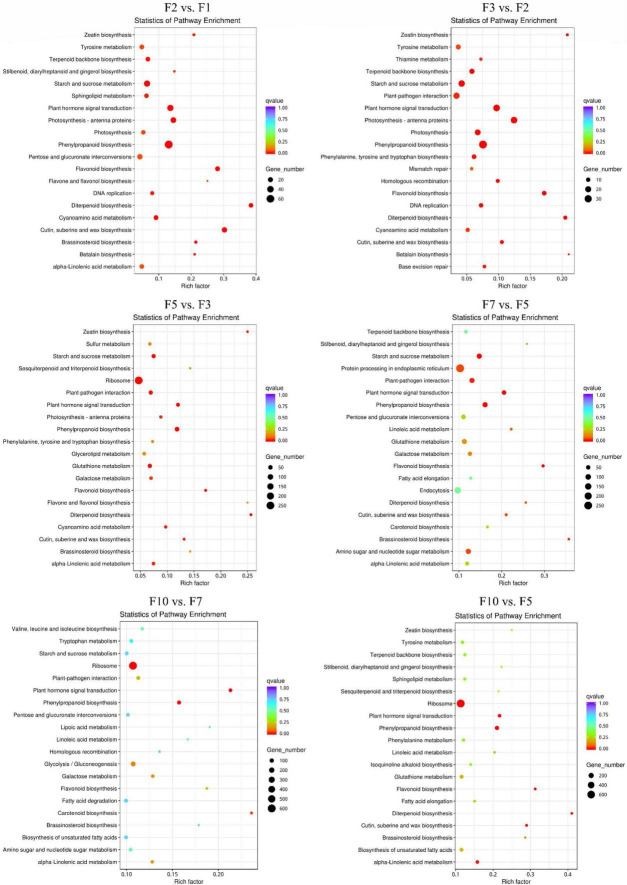
Scatter diagram of the enriched Kyoto encyclopedia of genes and genomes (KEGG) pathways in *Dacrydium pectinatum* female cones. F, female cones. 1: January, 2: February, 3: March, 5: May, 7: July, 10: October.

**FIGURE 8:**
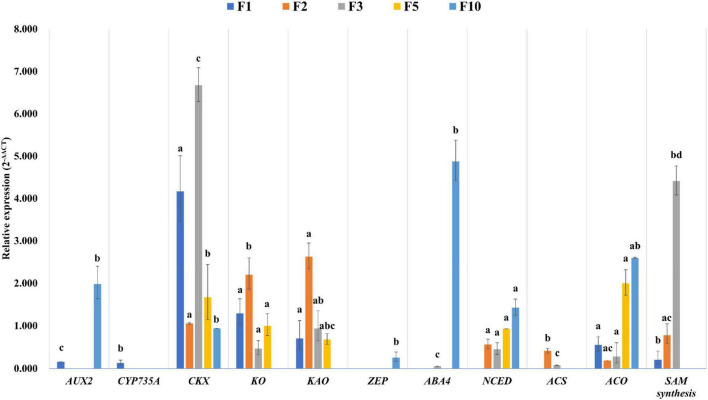
Reverse transcription-quantitative polymerase chain reactions (RT-qPCR) results of eleven unigenes related to endogenous hormone synthesis pathway. Elongation factor (EF1-α) gene was used as the reference gene for normalization of gene-expression data. All data are averages of three independent experiments, and error bars represent scanning electron microscopy (SEM). Bars with the same lowercase letters within a genotype indicate not significant differences by Fisher’s least significant difference test at *p* < 0.05.

When the timepoints were compared sequentially within the female cone samples, a total of 22,559 unigenes were differentially expressed (Figure 6D). These DE unigenes were mapped to 308 KEGG pathways with 20 pathways being significantly enriched in F2 vs. F1, F3 vs. F2, F5 vs. F3, F7 vs. F5, F10 vs. F7, and F10 vs. F5, respectively (Figure 7). Among these significant pathways, starch and sucrose metabolism, plant hormone signal transduction, phenylpropanoid biosynthesis, and flavonoid biosynthesis occurred in all five comparisons, while diterpenoid biosynthesis, cutin, suberine, and wax biosynthesis, brassinosteroid biosynthesis, and plant-pathogen interaction were found in four comparisons. When verified with RT-qPCR, 9 out of 11 comparisons had the same regulated trend as the RNA-Seq data (Figure 8), indicating our RNA-Seq reflected the relative differences in gene expression.

### Differentially expressed *Dacrydium* unigenes involved in hormone metabolism and hormone signal transduction pathway during female cone development

Comparisons of F2 vs. F1, F3 vs. F2, F5 vs. F3, and F10 vs. F5 revealed a set of 15, 17, 20, 10, and 15 DE unigenes that are involved in IAA, CTK, GA, ABA, and ethylene biosynthetic and metabolic pathways (Table 1). These unigenes encode for 26 different genes; 20 genes are in the biosynthesis pathway of endogenous hormones, except 6 genes for *DAO*, *IAMT1*, *GH3*, *APRT*, *CKX*, and *2ox* are in the metabolism pathway (Supplementary Figure 6). GA had the most DE unigenes (20), followed by CTK (17).

**TABLE 1:** Differentially expressed *Dacrydium pectinatum* unigenes involved in hormone biosynthetic and metabolic pathways among female cone samples.

Hormone name	F2 vs. F1		F3 vs. F2		F5 vs. F3		F10 vs. F5		Total DE unigenes	Total annotated genes
	Up	Down	Up	Down	Up	Down	Up	Down		
IAA	5	6	4	2	2	4	5	6	15	**8** (Anthranilate phosphoribosyltransferase; Auxin transporter-like protein 1; Indoleacetamide hydrolase; Indole 3 acetamide hydrolase; Methylindole-3-acetic acid 1; 2-oxoglutarate-dependent dioxygenase; Indole-3-acetate O-methyltransferase 1; Indole-3-acetic acid-amido synthetase)
CTK	2	4	4	1	5	2	4	5	17	**5** (Cytokinin *trans*-hydroxylase; Cytokinin riboside 5′-monophosphate phosphoribohydrolase; Adenine phosphoribosyltransferase; Cytokinin dehydrogenase; Adenylate kinase)
GA	7	7	5	3	2	8	7	9	20	**5** (*ent*-Copalyl diphosphate synthase; *ent*-Kaurene oxidase; *ent*-Kaurenoic acid oxidase; Gibberellin 20 oxidase; Gibberellin 2 oxidase)
ABA	0	2	1	0	2	1	4	2	10	**5** (Zeaxanthin epoxidase; 9-*cis*-epoxycarotenoid dioxygenase; Molybdenum cofactor sulfurase; Short-chain dehydrogenase/reductase; Violaxanthin de-epoxidase)
Ethylene	9	1	2	1	3	5	3	5	15	**3** (1-aminocyclopropane-1-carboxylate synthase; 1-aminocyclopropane-1-carboxylate oxidase; L-methionine-S-adenosyltransferase)

The differentially expressed unigenes related to endogenous hormone biosynthesis and metabolism pathway obtained by RNA-Seq were verified by RT-qPCR (Figure 8). The expression of the *AUX2* gene was higher in the female cone in October at the ovule maturation stage. *CKX* was highly expressed in the early stage of bract primordium differentiation of *D. pectinatum* in March. The expression of *KO* and *KAO* in the GA biosynthesis pathway was the highest at the flower bud differentiation stage in February. The *ABA4* and *NCED* genes in the ABA biosynthesis pathway were highly expressed in the ovule maturation stage in October. In addition, the expression of *ACO* also peaked in October, while the *SAM synthesis* gene in the ethylene biosynthesis pathway was highly expressed in March.

A set of 25, 2, 6, 14, and 8 DE unigenes are involved in the IAA, CTK, GA, ABA, and ethylene signal transduction pathways, respectively, representing 5, 1, 4, 4, and 7 annotated genes (Table 2). Most of these unigenes (25) are involved in the IAA signal transduction, followed by ABA (14) and ethylene (8). The up- and downregulated numbers were 41 and 46, respectively. Annotations of these unigenes are listed in Supplementary Table 4.

**TABLE 2:** Differentially expressed *Dacrydium pectinatum* unigenes involved in hormone signal transduction pathway among female cone samples.

Hormone name	F2 vs. F1		F3 vs. F2		F5 vs. F3		F10 vs. F5		Total DE unigenes	Total annotation categories
	Up	Down	Up	Down	Up	Down	Up	Down		
IAA	5	8	7	2	1	8	6	8	25	5
CTK	0	0	0	1	0	0	1	0	2	1
GA	1	2	1	0	2	1	2	1	6	4
ABA	1	1	0	2	2	3	7	4	14	4
Ethylene	2	1	0	2	1	1	2	1	8	7
Total	9	12	8	7	6	13	18	14	55	21

### Differentially expressed unigenes involved in the development of reproductive structures

**FIGURE 9:**
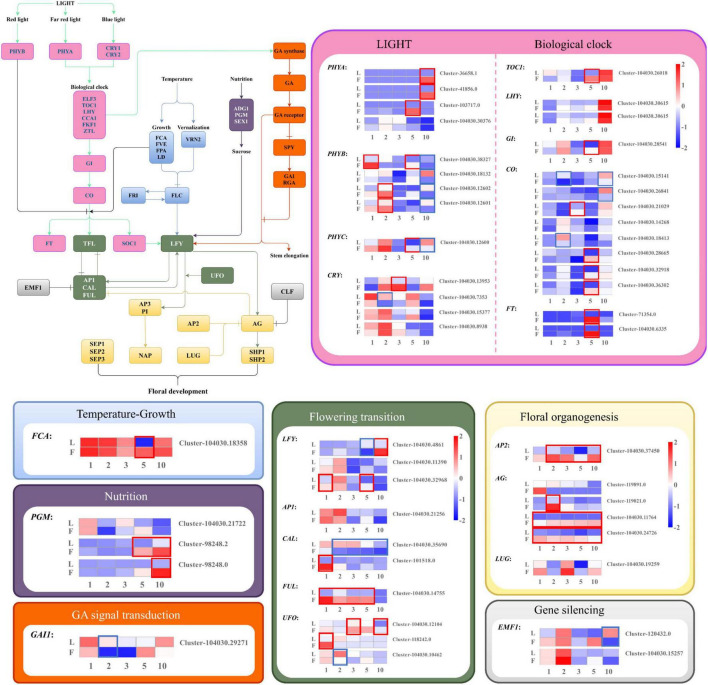
Differentially expressed flowering *Dacrydium pectinatum* unigenes identified in comparisons between leaf and female cone tissues. The gradient-colored barcode at the top right indicates the log_2_(FC) value. Fold change is calculated based on the difference multiple of fragments per kilobase of transcript per million mapped reads (FPKM) in different periods. The unigenes expression in female cone was significantly higher than leaves, which was framed in red box, and lower was in blue box. 1, 2, 3, 5, and 10: January, February, March, May, and October 2019; L, leaves; F, female cones. PHYA/B, Phytochrome A/B; CRY1/2, Cryptochrome 1/2; ELF3, Early flowering 3; TOC1, Timing of CAB1; LHY, Late elongated hypocotyl; CCA1, Circadian clock-associated 1; FKF1, Flavin-binding kelch repeat F-box 1; ZTL, Zeitlupe; GI, Gigantea; CO, Constans; FT, Flowering locus T; SOC1, Suppressor of constants overexpression 1; FCA, Flowering control local A; FVE: MSI1-homolog, (a conserved WD-repeat protein found in many chromatin complexes); FPA, RRM-domain proteins; LD, Luminidependens; VRN2, Vernalization 2; FLC, Flowering locus C; FRI, Frigida; ADG1, ADP glucose pyrophosphorylase; PGM, Phosphoglucomutase; SEX1, Starch excess 1; GA, gibberellin; SPY, Spindly; GAI, Gibberellic acid insensitive; RGA, Repressor of GA; TFL, Terminal flower 1; LFY, Leafy; AP1/2/3, Apetala 1/2/3; CAL, Cauliflower; FUL, Fruitfull; UFO, Unusual floral organs; EMF1, Embryonic flower 1; CLF, Curly leaf; PI, Pistillata; AG, Agamous; SEP1/2/3, Sepallata1/2/3; NAP, NAC-like activated by AP3/PI; LUG, Leunig; SHP1/2, Shatterproof 1/2.

When compared to leaf samples at the same timepoints, a set of 21 genes in the flower development pathway was differentially expressed, with 29 upregulation unigenes and 14 downregulation unigenes (Figure 9). Nine of these genes were found in the photoperiod pathway (light), one in the growth (temperature) pathway, one in the nutrition pathway, one in the GA pathway, while five were associated with floral integrator genes and three were with floral organ identity genes. Notably, compared with leaf tissue, all *PHYB* unigenes were downregulated in October. In the biological clock pathway, all genes except the *LHY* gene were upregulated in May, and the *FCA* gene with promoting effect was also upregulated in May. In the flowering transition pathway, *CAL* and *FUL* exhibited the opposite regulation in February, March, and May.

**FIGURE 10:**
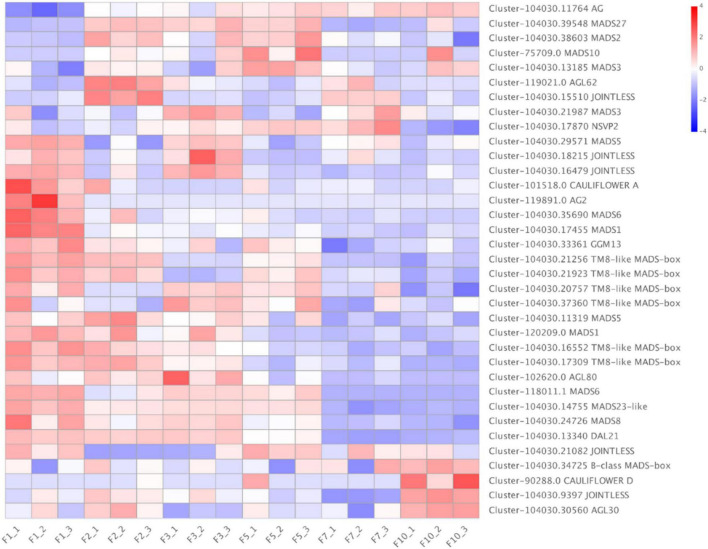
Expression dynamics of *Dacrydium pectinatum* MADS-box unigenes differentially expressed during female cone development. The gradient-colored barcode at the top right indicates the log_2_(FC) value. Fold change is calculated based on the difference multiple of fragments per kilobase of transcript per million mapped reads (FPKM) in different periods. The unigenes expression in female cone was significantly higher than leaves, which was framed in red box, and lower was in blue box. 1, 2, 3, 5, and 10: January, February, March, May, and October 2019; F, female cones. Each group contains three repeats. AG, Agamous; AGL, Agamous-like MADS-box protein.

Among the 35 DE MADS-box unigenes, *MADS2* were highly expressed in February and May, and *MADS1, AGL62* were highly expressed in January and February female cones, respectively (Figure 10). There were 20 MADS-box unigenes with the highest expression in the January female cones. One each *B-class MADS-box*, *CAULIFLOWER D, JOINTLESS*, and *AGL30* unigenes were exclusively expressed in samples in October.

## Discussion

### Development of *Dacrydium pectinatum* female cone lasts for 2 years and its single grow at the top of branches

In addition to one nothospecies, the genus *Dacrydium* also includes 21 species.^3^ The distribution and ecology of *Dacrydium* extends from New Zealand in the south through the islands of New Caledonia, Fiji, the Solomons, New Guinea, and Indonesia to the Philippines, Thailand, and southern China in the north (Quinn, 1982). Currently, not much information is available about when the initiation occurs for reproductive cones in a *Dacrydium* species. In New Zealand, the *D. cupressinum* cone initiation is suggested to occur in late summer or autumn with pollination occurring in spring (Norton et al., 1988). Our morphohistological study indicates that the *D. pectinatum* female cones initiate before mid-January in Hainan Island, China, because female buds are distinguishable by late January (Figures 1A, 3A). Compared with other coniferous species, a relatively high level of genetic variation and a low degree of differentiation were revealed in *D. pectinatum* (Su et al., 2010), which can be the contributing factors to the discrepancy observed. Mature *D. pectinatum* female cones average 7.35 mm in length and the diameter reaches 4.57 mm (Figure 1B), similar to de Laubenfels’s report for the same species (De Laubenfels, 1988). When more information becomes available for other *Dacrydium* species, it will be interesting to compare their reproductive buds and cones’ morphology and phenology. This will help understand the diversity among the genus.

Similar to other conifer species, such as *Metasequoia glyptostroboides*, the female cones of *D. pectinatum* are covered with ovule scales. However, unlike the female cone scales and leaves decussately arranged in *M. glyptostroboides*, the female cones of *D. pectinatum* are only single grow at the top of branches (Miki, 1941). The female cones are mainly located around the outer and sunlit parts of the crown. This is advantageous for pollen dispersal by wind, which is common in conifers (Leslie, 2011a,b). Our study suggests that the development of *D. pectinatum* megaspore can be divided into six stages (Figures 1–3): early flower bud differentiation (January), bract primordium differentiation (March and April), ovule primordium differentiation (June), dormancy (November to January of the second year), ovule maturity (August of the second year), and seed maturity (November and December of the second year). This process lasts for about 24 months. During March and April of the first year of female cone development, the male cone pollen begins to spread. At this time, the ovule scale of the female cone opens, and the pollen fall on the micropyle and are adhered by the exuded colloidal liquid. Then the ovule scale closes, and after the colloidal liquid dries, the pollen contacts the nucellus, sprout short pollen tubes, and enter the dormancy period. The long development period can arguably be a contributing factor to the low seed quality of the species, considering that many adverse factors such as extreme weather and diseases can occur, affecting the quality and quantity of pollen production. We will conduct a similar study on female and male cones including all months shortly. The new information will further reveal the reproductive system development mechanism of the species.

### Endogenous hormones fluctuate during the process of female cone development

Plant hormones play an irreplaceable role in regulating plant growth, development, differentiation, and reproduction. The major phytohormones include auxins, abscisic acid, cytokinin, ethylene, gibberellin, brassinosteroids, jasmonates, and strigolactones (Davis, 2009; Kazan and Lyons, 2016). The effects of hormones and their balance on reproductive bud initiation and development seem to depend on species and sex. GA_4+7_ is more beneficial to induce conifers to produce microsporophylls (Pharis et al., 1980; Sheng and Wang, 1990). CTK promots the development of female cones in *Mercurialis leiocarpa* Sieb. et Zucc. and *Vitis amurensis* Rupr. (Nelson and Irish, 1989). IAA promots the development of female inflorescence, and endogenous ABA and GA_3_, ZR and IAA, ABA and IAA all have obvious antagonistic effects, while ZR and GA_3_ show synergistic effects (Lan et al., 2018). Low levels of GA are reported to be beneficial for female cone formation in *Pinus* (Niu et al., 2014) and other conifers, such as Douglas-fir (Kong et al., 2008). GA_4/7_ promotes female flowering in black spruce, but inhibits it in white spruce (Greenwood et al., 2011). In our previous study with *Metasequoia*, higher levels of IAA and GA_1+3_ and a lower level of ABA were beneficial to female primordium initiation, while higher levels of GA_1+3_ and lower levels of IAA and ABA were favorable to male cone initiation (Liang and Yin, 1994). In *D. pectinatum*, the level of GA, IAA, ABA, and CTK fluctuated during the process of female cone development, suggesting that different development stages require various dosages and interplay of endogenous hormones (Figure 4). Our data corroborate the findings in lodgepole pine (Kong et al., 2012).

Abscisic acid is involved in maintaining seed dormancy in plants. The gymnosperm seeds need to be frozen to reduce ABA content for germination (Schmitz et al., 2002; Feurtado et al., 2004, 2007; Liu et al., 2015). Remarkably, seed germination of gymnosperms depends not only on ABA-GA homeostasis but also on IAA-ABA homeostasis (Liu et al., 2015). In this study, the content of GA_3_ was at a high level when *D. pectinatum* was in dormancy, and the contents of IAA and ABA were higher than in the mature stage. In the second year, it seems to need to maintain a low level of GA_3_-IAA-ABA steady state to promote seed germination (Figure 4). This provides new insights for improving the seed germination rate of *D. pectinatum*.

### Ribonucleic acid sequencing reveals many *Dacrydium* genes

A search of “*Dacrydium*” in the NCBI databases resulted in only 305 nucleotide sequences as of May 2022. Almost all these sequences either belong to the chloroplast genome or are microsatellite sequences. However, the transcriptome data of male *D. pectinatum* (accession number: PRJNA748147) that revealed > 70,000 unigenes uploaded by NCBI in 2021 have not been disclosed yet (Lu et al., 2021). Therefore, nearly all of the > 184,000 unigenes generated from our RNA sequencing of 36 samples were revealed for the first time in female *D. pectinatum*. It fills the gap in the genomic resources of female *D. pectinatum*. To our knowledge, this is the only female *Dacrydium* transcriptome being reported. When better characterized and combined with male transcriptome analysis, the unigenes can be applied to revealing the molecular mechanisms of various important biological processes in *Dacrydium*. In further understanding the characteristics of the *D. pectinatum* genome, we found that the GC content of the female *D. pectinatum* transcriptome was 44.99%, the same as the male transcriptome (45%) and in addition, similar to what has been reported in other conifer species, e.g., 47% in *Amentotaxus argotaenia* (Ruan et al., 2019) and 44.58% in *Pinus dabeshanensis* (Xiang et al., 2015). The dominant SSR type was mononucleotide repeats with 9–12 bps both in female and male *D. pectinatum*.

### Gene expression is modulated during *Dacrydium pectinatum* female cone development

A total of 95,510 differentially expressed unigenes were found between leaves and female cones (Figure 6A). Since a separate study is undertaken to compare female cones and leaves in terms of morphology, histology, hormone dynamics, and gene expression, and previous studies have described the development of male cones. So, we focused on the development of female cones in this study. The M10 (female cones of October) vs. F5 (female cones of May) comparison generated the most DE unigenes (13,594). These two timepoints reflect the transition from the ovule development stage to the seed maturation stage and experience the key stage of fertilization (Figure 3). The fact that hormone signal transduction was found to be significantly enriched in the F10 vs. F5 comparison (Figure 7) underscores the importance of hormones in this transition. When genes in the endogenous hormone-related pathways were examined, 77 *D. Pectinatum* DE unigenes were found in the biosynthesis and metabolism process, and 55 DE unigenes were found in the signaling pathway. This suggests that the observed fluctuations in hormone levels during female cone development are mainly due to changes in their synthesis and degradation. The expression of genes related to the hormone signal transduction pathway also changes with the fluctuation of hormone content. Since 16 of the 26 DE hormone biosynthesis and metabolism genes contain multiple unigenes with different expression patterns (Supplementary Figure 6), further research is warranted to better understand their roles. Considering that IAA maintained a higher level during female cones development when compared to the earliest timepoint (January) and had the most DE-annotated genes in both hormone anabolism and signal transduction pathways, IAA may be effective in female cones induction in juvenile *D. Pectinatum* trees. After all, IAA has been applied to *Gnetum parvifolium* to promote female cones to shorten juvenility (Lan et al., 2018).

Of the 21 DE flowering gene homologs identified among comparisons of female cone samples (Figure 9), nine of these genes were found in the photoperiod pathway, one in the growth pathway, one in the nutrition pathway, one in the GA pathway, five were associated with floral integrator genes, and three were with floral organ identity genes, suggesting the importance of these pathways in the female cone initiation and development. Several DE MADS-box homologs of the timepoint-specific unigenes were found to be exclusively expressed in the female reproductive part in other species. Include *MADS2* was expressed in high abundance in the whole period female cones of *Pinus tabuliformis* (Niu et al., 2022) and *AGL62* was revealed to be active in the female gametophyte or developing seed in *Arabidopsis* (Bemer et al., 2010). In comparison, the *D. pectinatum MADS2* and *AGL62* homologs were found expressed in an earlier flower bud differentiation stage (Figure 10). Furthermore, *DAL11*/*12*/*13* and *MADS43*/*45* are also specifically expressed in female cones in *Pinus tabuliformis* (Niu et al., 2016), and B-sister genes are expressed only in female reproductive tissues in *Picea abies* (Rudall et al., 2011). *JOINTLESS* is a MADS-box gene controlling flower abscission zone development (Mao et al., 2000; Pineda et al., 2010). Thus, it is reasonable to see its expression in the October samples when female cones start to reach the seed maturation stage. In November, the seeds gradually mature and fall off. The six *TM8-like MADS-box D. pectinatum* homologs were largely highly expressed in the January samples, suggesting their roles in the megaspore primordium formation. The function of *TM8-like MADS-box* genes remains to be demonstrated. The *TM8* gene was defined as an “early” gene in *Arabidopsis* (Pnueli et al., 1991). The “early” *TM8* genes are quite common also in gymnosperms and might in some yet unknown way regulate the expression of the “late” genes (Daminato et al., 2014; Gramzow et al., 2014).

The comparisons of female cones with leaves at the same timepoints also identified the photoperiod pathway having the most DE unigenes (Figure 9), further suggesting its importance in the female cone initiation and development of *D. pectinatum*. *TOC1*, *GI*, *CO*, and *FT* genes in the biological clock pathway were highly expressed in the early ovule development stage of female cones indicating that the biological clock pathway is essential for the initiation of the ovule. In consistence with LFY’s essential roles in floral meristem identify and organ identify, two of the three *D. pectinatum LFY* unigenes were upregulated across January and February being surveyed. Considering that GAI is a repressor of GA responses and inhibits the expression of *LFY*, the results of *GAI* being upregulated in May and October that GA inhibitors such as paclobutrazol (PBZ) can be applied to induce cone mature in *D. pectinatum*. Currently, PBZ is effective in promoting flowering in some woody species such as camellia, apple, and mango (Wei et al., 2017, 2018, 2021; Wongsrisakulkaew et al., 2017; Sha et al., 2021). The high expression of *PGM* in October is consistent with the rapid accumulation of nutrients by the seeds at maturity.

## Conclusion

The paucity of basic research and knowledge on *D. pectinatum* is a challenge for us to protect and reproduce endangered gymnosperms. In this study, we reported that the female flower buds of *D. pectinatum* began to differentiate in January of the first year and the seeds matured in December of the second year in the mountainous tropical rain forest of Hainan, China. The dynamic change of endogenous hormones suggests their roles in different stages of cone development. Hormone-induced female cones can provide an alternative method to solve the lack of natural regeneration caused by the long juvenility and poor seed quality of the species. It is suggested that the IAA analogs should be sprayed on the top of branches no later than January before the initial differentiation of reproductive buds to induce the initiation of female flower buds. PBZ is sprayed before May to induce early maturation of ovules, adjust to the same period as pollen dissemination, and improve the fertilization rate. The RNA sequencing analyses generated the first transcriptome database for female *Dacrydium* and revealed several floral (*MADS2*, *AGL62*, and *LFY*) and hormone biosynthesis and signal transduction (*CKX*, *KO*, *KAO*, *ABA4*, *ACO*, etc.) genes that could be critical for female cone development. Large-scale and complete transcriptome resources of *D. pectinatum* will broaden the bottleneck of genetic and genomic research in this genus and push the research on the development mechanism of male and female cones of gymnosperms to the “fast lane” of omics. Our study is of significance, representing a starting point for in-depth analyses of the species’ reproductive development that will help tackle the challenges of low seed quality and poor natural regeneration.

## Data availability statement

The data presented in this study are deposited in the NCBI repository, accession number PRJNA842859.

## Author contributions

YZ had the main responsibility for data collection and analysis and had the overall responsibility for experimental design, and manuscript preparation. WL conducted the records of anatomy and endogenous hormones of female cones experiments. EW and XZ conducted qRT-PCR. YZ and WL performed the RNA-Seq. EW, HL, SH, XS, and YZ analyzed the data. EW drafted the manuscript which was critically revised by YZ. All authors have read and approved the final manuscript.

## Footnotes

1
https://www.iucnredlist.org/

2
http://trinityrnaseq.sf.net

3
https://www.conifers.org/po/Dacrydium.php
