# Supplementary figures and images for: Morpho-histology, endogenous hormone dynamics, and transcriptome profiling in Dacrydium pectinatum during female cone development
Source: Front Plant Sci. 2022 Aug 17;13:954788. doi: 10.3389/fpls.2022.954788 (PMC9428629; doi:10.3389/fpls.2022.954788)

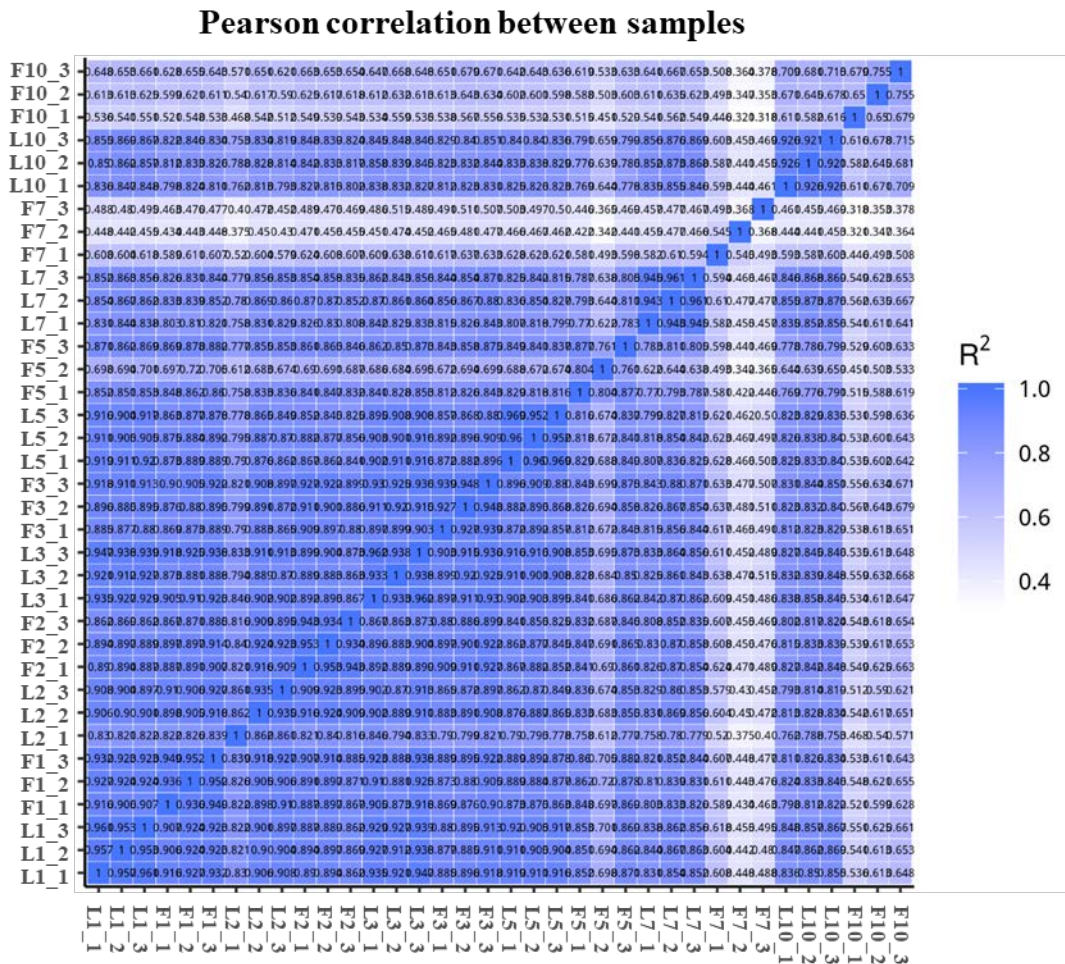

**Supplementary Figure 1.** Pearson correlation of the *D. pectinatum* RNA-seq samples.

Supplement: Supplementary file 1 [file Data_Sheet_1.PDF]

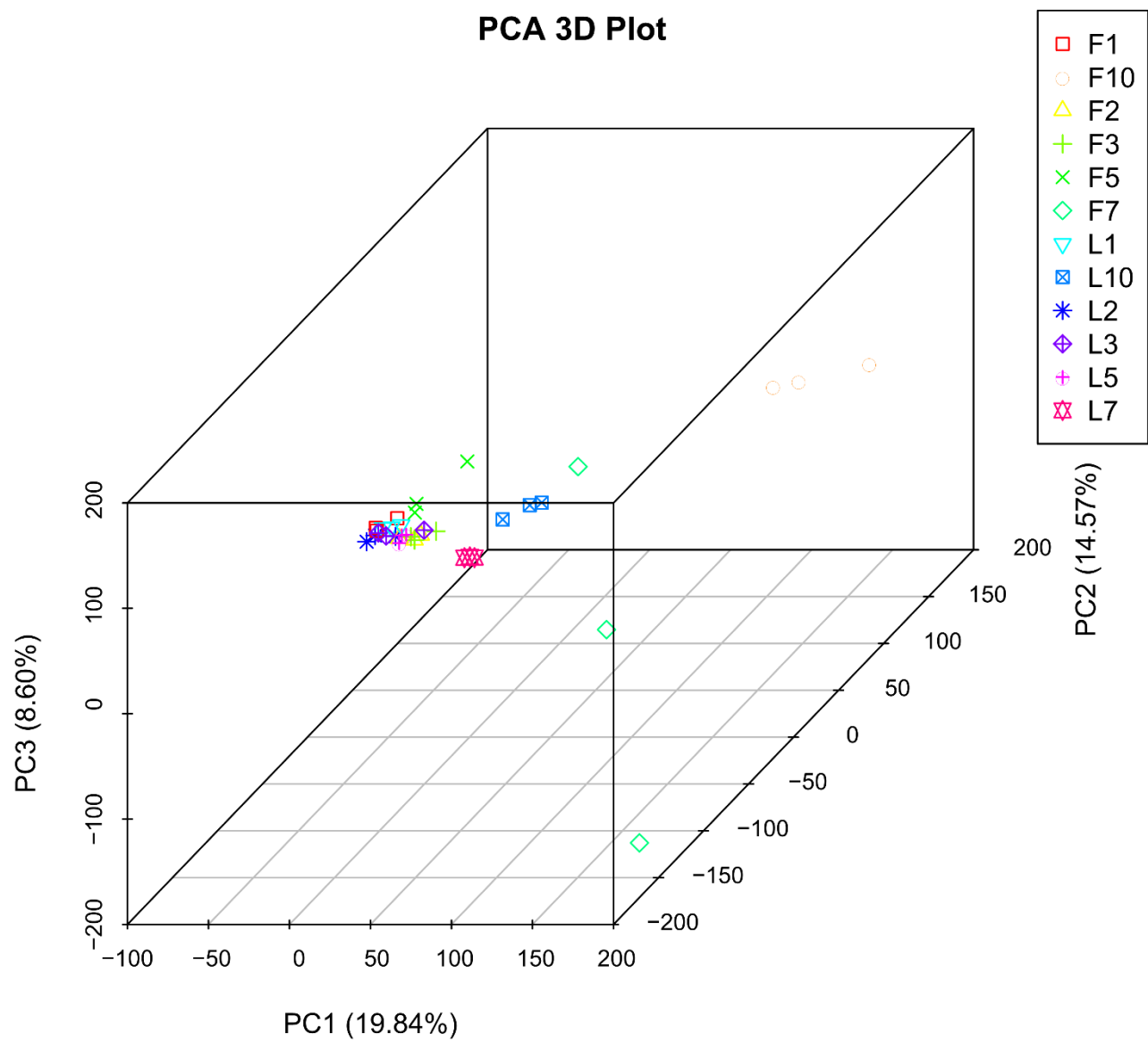

**Supplementary Figure 2.** Principal component analysis of the *D. pectinatum* RNA-seq samples.

Supplement: Supplementary file 2 [file Data_Sheet_2.PDF]

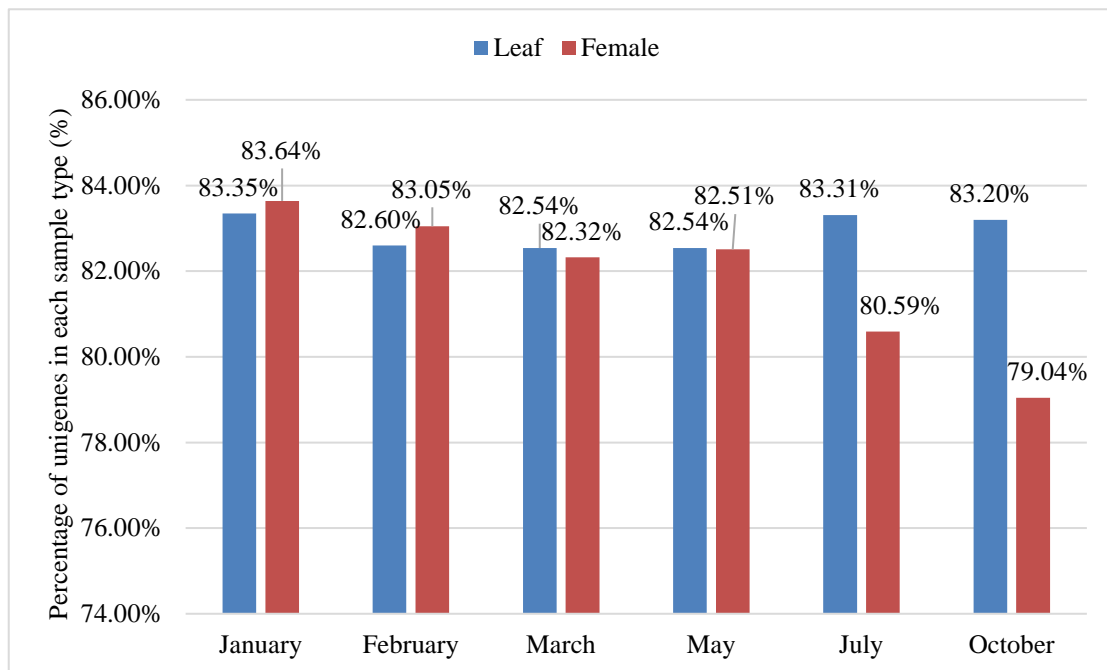

**Supplementary Figure 3.** Percentage of unigenes in each *Dacrydium pectinatum* sample type.

Supplement: Supplementary file 3 [file Data_Sheet_3.PDF]

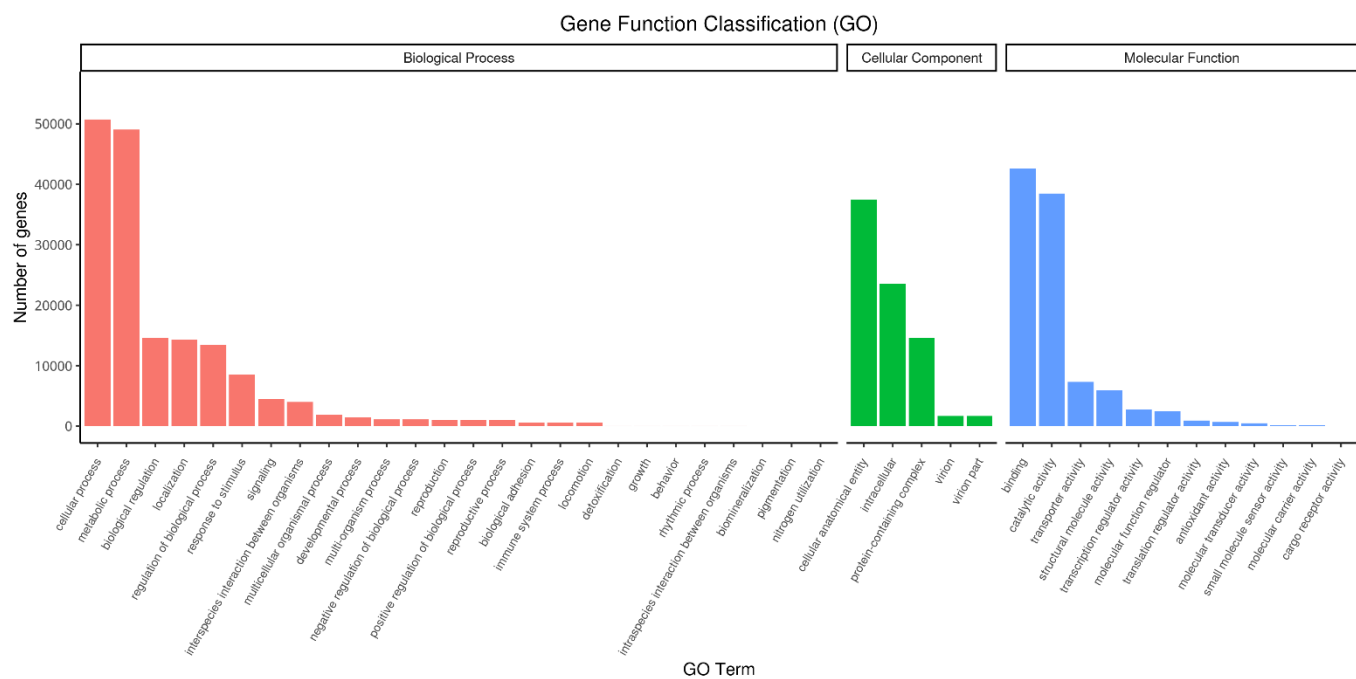

**Supplementary Figure 5.** GO annotation of *Dacrydium pectinatum* unigenes.

Supplement: Supplementary file 5 [file Data_Sheet_5.PDF]
