# Supplementary material for: Morpho-histology, endogenous hormone dynamics, and transcriptome profiling in Dacrydium pectinatum during female cone development
Source: Front Plant Sci. 2022 Aug 17;13:954788. doi: 10.3389/fpls.2022.954788 (PMC9428629; doi:10.3389/fpls.2022.954788)
Supplement: Supplementary file 6 [file Data_Sheet_6.PDF]

A. Indoleacetic acid (IAA)

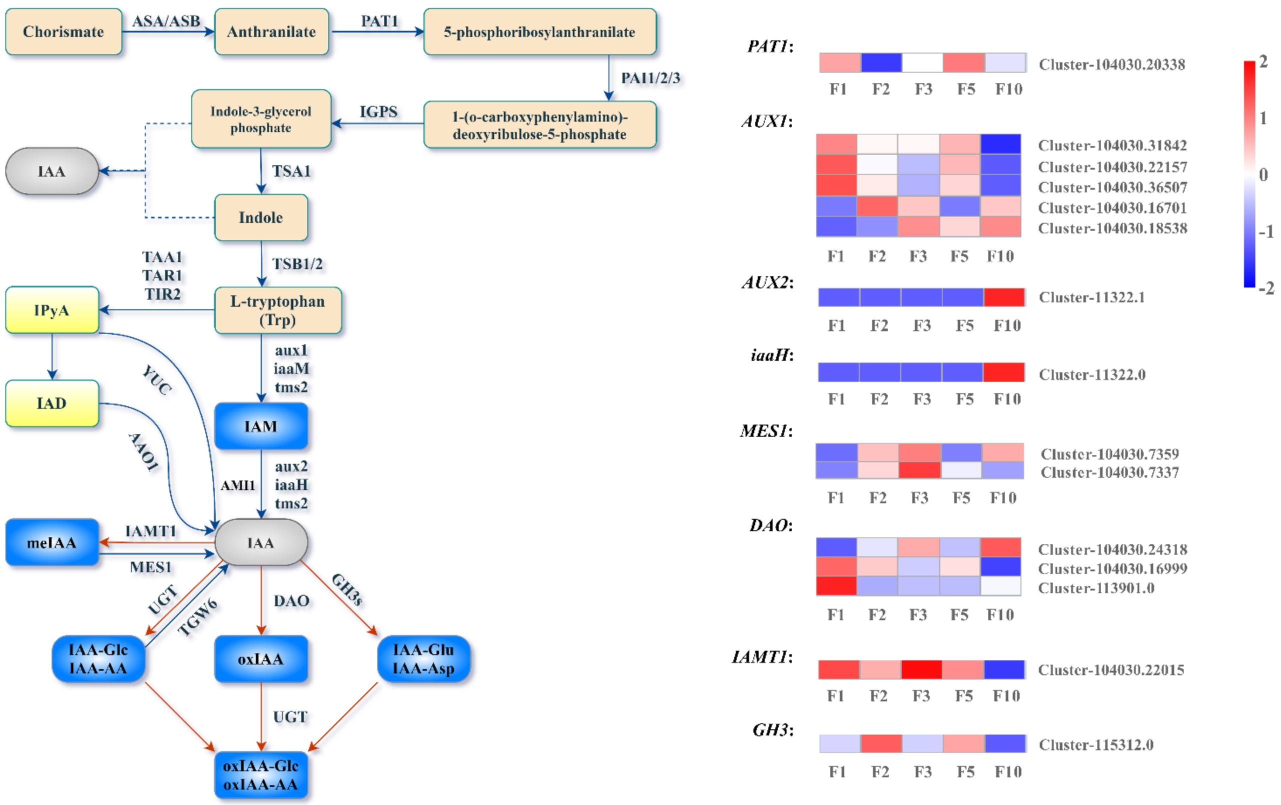

B. Cytokinin (CTK):

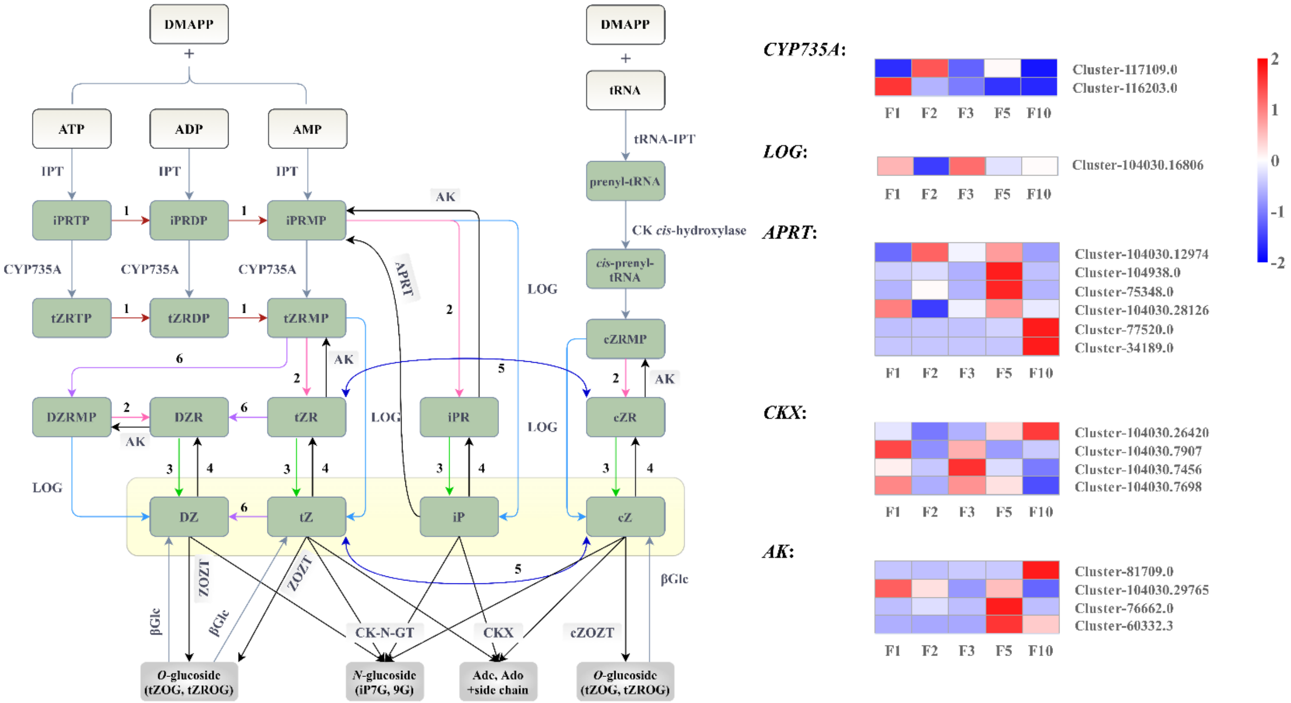

C. Gibberellin (GA)

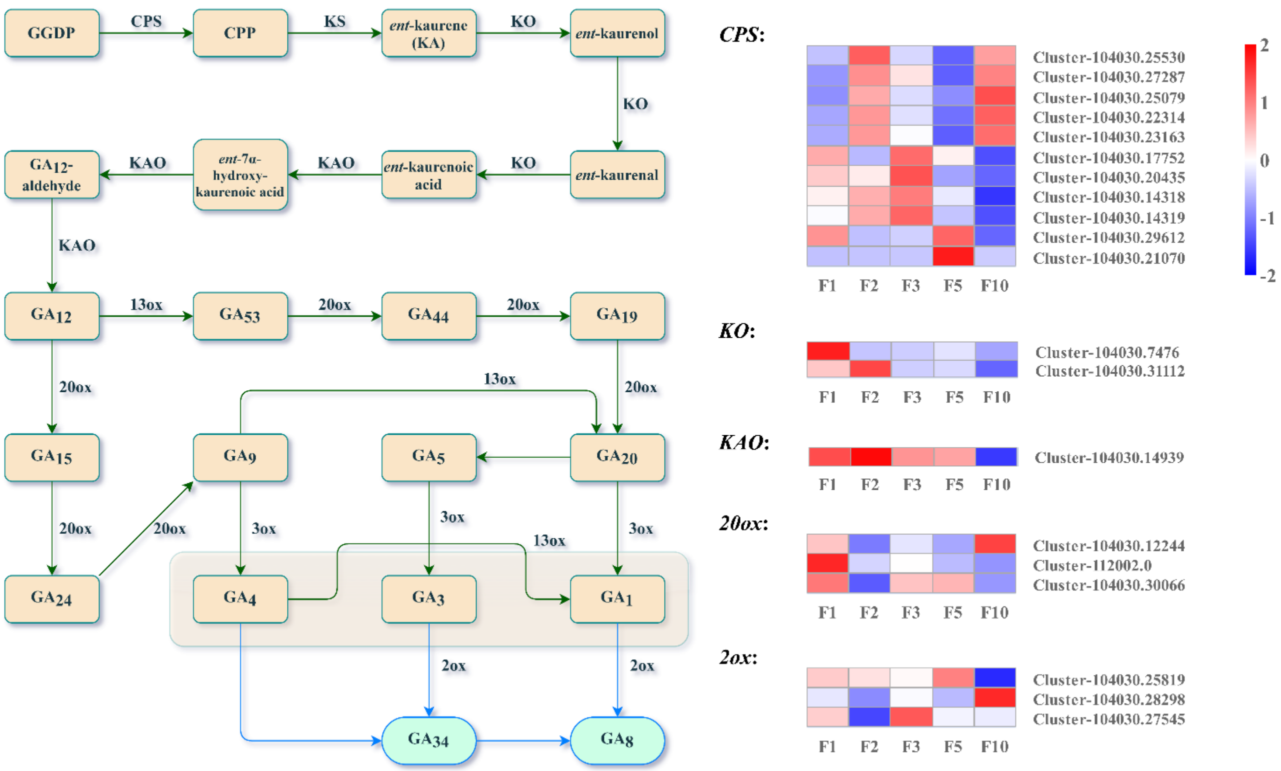

D. Abscisic acid (ABA)

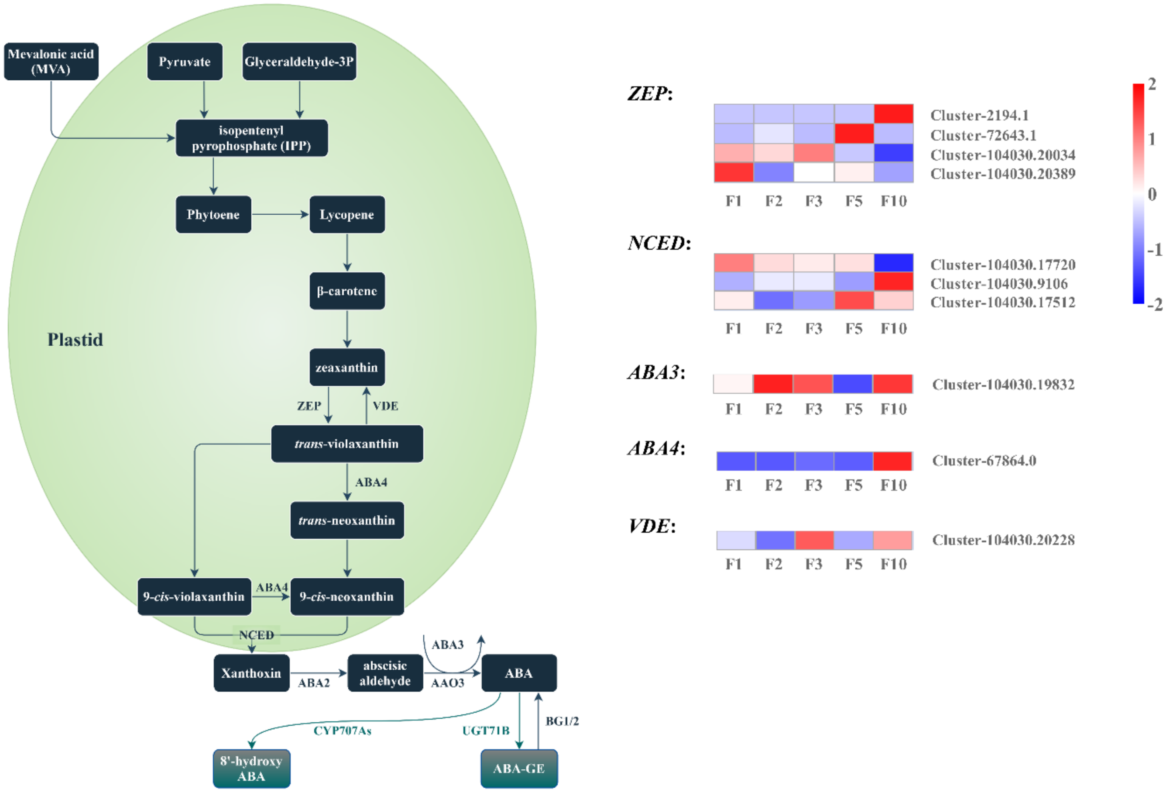

E. Ethylene:

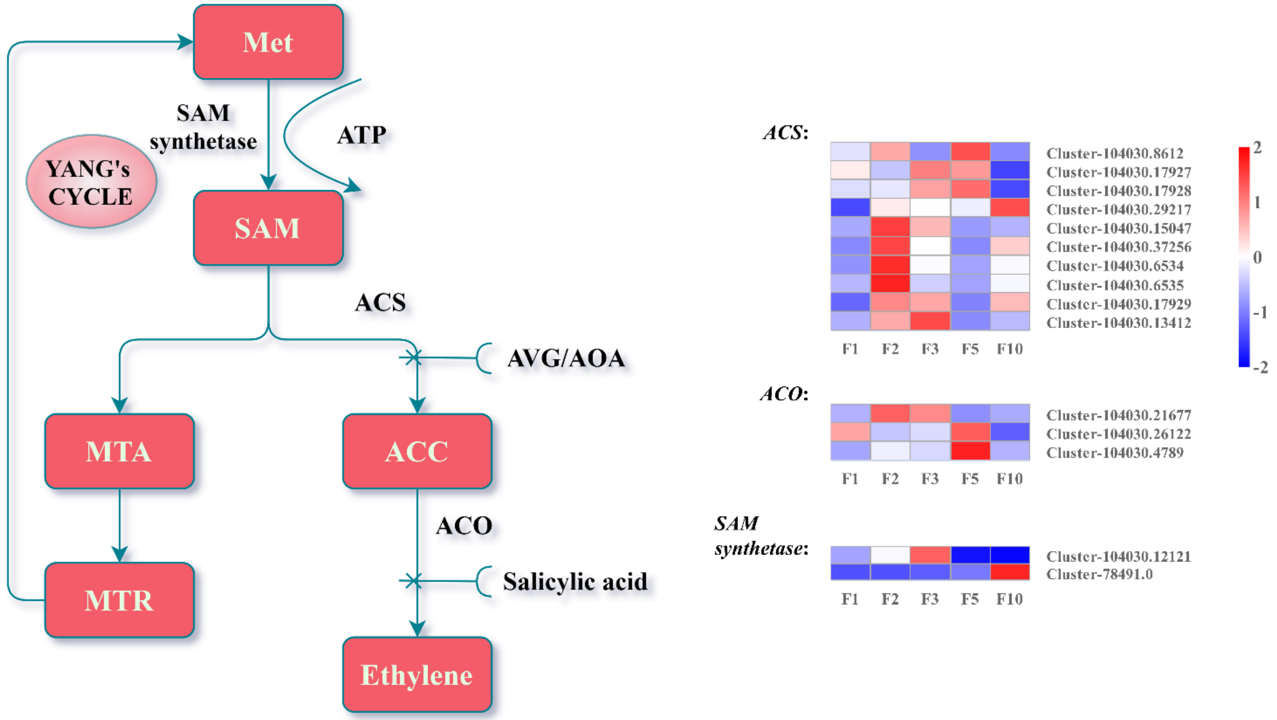

Supplementary Figure 6. Differentially expressed *Dacrydium pectinatum* unigenes identified in hormone metabolic pathways.

Cluster analysis of differentially expressed unigenes identified in *D. pectinatum* female cone of various development stages (F1, 2, 3, 5, 10: January, February, March, May and October 2019). The gradient-colored barcode at the top right indicates the $\log_2$(FC) value. Fold change is calculated based on the difference multiple of FPKM in different periods. **A:** Presumptive pathways for indole-3-acetic acid biosynthesis and metabolic in plants. A dashed arrow denotes the tryptophan-independent IAA biosynthetic pathway. IAA: Indole-3-acetic acid; IAM: Indole-3-acetamide; IPyA: Indole-3-pyruvic acid; IAD: Indole-3-acetaldehyde; meIAA: Methylindole-3-acetic acid; oxIAA: 2-oxindole-3-acetic acid; IAA-Glc: IAA-glucose; IAA-AA: IAA-amino acid; IAA-Glu: IAA-glutamic acid; IAA-Asp: Aspartic acid; ASA: Anthranilate synthase α subunit; ASB: Anthranilate synthase β subunit; PAT: phosphoribosylanthranilate transferase; PAI: phosphoribosylanthranilate isomerase; IGPS: Indole-3-glycerol phosphate synthase; ISA: Trp synthase α subunits; TSB: Trp synthase β subunits; TAA1/TAR1/TIR2: Tryptophan aminotransferase; YUC: YUCCA flavin-containing monooxygenases; AAO1: Aldehyde oxidase; aux1/2: Auxin transporter-like protein 1/2; AMI1: Amidase-like protein 1; IAMT1: Indole-3-acetate O-methyltransferase 1; MES1: Methylesterase 1; DAO: Dioxygenase for auxin oxidation; UGT: Uridine-diphosphate glycosyltransferase; GH3: Gretchen hagen3; TGW6: IAA hydrolases. **B:** A current model of cytokinin biosynthesis and the two known activation pathways. DMAPP: Dimethylallyl diphosphate; iPRTP: iP riboside 5'-triphosphate; iPRDP: iP riboside 5'-diphosphate; iPRMP: iP riboside 5'-monophosphate; tZRTP: tZ riboside 5'-triphosphate; tZRDP: tZ riboside 5'-diphosphate; tZRMP: tZ riboside 5'-monophosphate; DZRMP: DZ riboside 5'- monophosphate; cZRMP: cZ riboside 5'-monophosphate; DZR, DZ riboside; tZR: tZ riboside; cZR: cZ riboside; iPR: iP riboside; tZ: Cytokinins trans-zeatin; iP: N^6-(D2-isopentenyl) adenine; cZ: *cis*-zeatin; DZ: Dihydrozeatin; 1: tZRDP tZR 5-diphosphate; 2: tZRTP tZR 5-

triphosphate; 3: 5-ribonucleotide phosphohydrolase; 4: Adenosine nucleosidase; 5: Purine nucleoside phosphorylase; 6: Zeatin reductase; 7: Cytokinin *cis*-hydroxylase; IPT: Adenosine phosphate-isopentenyltransferase; CYP735A: Cytochrome P450 735A mono-oxygenases; LOG: Cytokinin nucleoside 5'-monophosphate phosphoribohydrolase; APRT: Adenine ribosyltransferase; AK: Adenylate kinase; CKX: Cytokinin oxidase; ZOGT: Zeatin *O*-glucosyltransferase; CK-N-GT: Cytokinin *N*- glucosyltransferase; βGlc: β-glucosidase. **C:** Unified gibberellins biosynthesis pathway. Bioactive GAs are shown in a yellow box. GGDP: geranylgeranyl diphosphate; CPP: *ent*-copalyl diphosphate; CPS: *ent*-Copalyl diphosphate synthase; KS: *ent*-Kaurene synthase; KO: *ent*-Kaurene oxidase; KAO: *ent*-kaurenoic acid oxidase; 13ox: 13 oxidase; 20ox: 20-oxoglutarate-dependent dioxygenase; 3ox: 3 oxidase; 2ox: 2 oxidase. **D:** Abscisic acid de novo biosynthesis occurs in the plastid and the cytosol. ABA is synthesized from the precursor isopentenyl diphosphate. The scheme shows the biosynthetic pathway from zeaxanthin to ABA. Enzymes are denoted next to the catalytic steps. ABA-GE: ABA-glucosyl ester; ZEP: Zeaxanthin epoxidase; VDE: Violaxanthin de-epoxidase; ABA4 is involved in the enzymatic step that catalyses the conversion of all-trans-violaxanthin into all- trans-neoxanthin; NCED: 9-*cis*-epoxycarotenoid dioxygenase; ABA2: Short-chain alcohol dehydrogenase/reductase; ABA3: Molybdenum cofactor sulfurase; AAO3: ABA-aldehyde oxidase 3; CYP707As: Cytochrome P450 707A family of proteins; UGT71B: ABA-uridine diphosphate glucosyltransferases 71B; BG1/2: β-glucosidase homologues 1/2. **E:** A current model of ethylene acid biosynthesis. Met: Methionine; SAM: S-adenosyl methionine; MTA: 5’-methylthioadenosine; MTR: Methylthioribose; ACC: 1-aminocyclopropane-1-carboxylic acid; SAM synthetase: L-methionine-S-adenosyltransferase; ACS: ACC synthase; ACO: ACC oxidase; AVG: Aminoethoxyvinylglycine; AOA: Aminooxyacetic acid.
