# Supplementary material for: Morpho-histology, endogenous hormone dynamics, and transcriptome profiling in Dacrydium pectinatum during female cone development
Source: Front Plant Sci. 2022 Aug 17;13:954788. doi: 10.3389/fpls.2022.954788 (PMC9428629; doi:10.3389/fpls.2022.954788)
Supplement: Supplementary file 7 [file Data_Sheet_7.PDF]

Supplementary Table 1. Sequences of primers used for RT-qPCR.

Gene ID	Forward Primer (5'-3')	Reverse Primer (5'-3')
Translation elongation factor (*EF1-α*)	GCAAGGAACTGGAGAAGGAACCC	GACGCATGTCCCTCACAGCAA
Cluster-11322.1 (*AUX2*)	AGTGGCTTCTGTTTCTGCTCG	AGGTTAATGACGTTGGTGTGA
Cluster-116203.0 (*CYP735A*)	AGGATATGACACTAGGGGAT	GATAAGATGGAGAGAGACGG
Cluster-104030.7456 (*CKX*)	AGCCCCGCGAAGAGTGAGAT	TCGTTGTTGAGGACCATGAA
Cluster-104030.31112 (*KO*)	TGGGAGAGAGGGGAGACGTT	GCGGGTTTTGATCTGGGAGT
Cluster-104030.14939 (*KAO*)	AGATCACGACAGGAGAAAA	GTCCAGCATTGAGATACAT
Cluster-2194.1 (*ZEP*)	TGAGAAAGCGTCGAACGACC	CTATCAGCATCCGCAATGAA
Cluster-67864.0 (*ABA4*)	TTGGAGGGAAAGACCTCTTC	CCGCCATCAACTTCGTAAAC
Cluster-104030.9106 (*NCED*)	AACCAAGCACAGAAATCCCC	TCCAATTCCGTCGTAGCCAA
Cluster-104030.6534 (*ACS*)	GAGGAGCCAACCCTAAAGGGA	TAGCGAAGCAGAATCTAAACC
Cluster-104030.4789 (*ACO*)	GGCCTTCAAGTGCTTAAAGA	ATAAAATGCTGCCACCGACA
Cluster-104030.12121 (*SAM synthesis*)	AGGAGGGTTTAATTGACGAAG	AAGAAGATGAGCGAAGGTGTG
