# Supplementary material for: Morpho-histology, endogenous hormone dynamics, and transcriptome profiling in Dacrydium pectinatum during female cone development
Source: Front Plant Sci. 2022 Aug 17;13:954788. doi: 10.3389/fpls.2022.954788 (PMC9428629; doi:10.3389/fpls.2022.954788)
Supplement: Supplementary file 8 [file Data_Sheet_8.PDF]

Supplementary Table 2. Summary of RNA sequencing data of *Dacrydium pectinatum* leave and Female cone tissues.

Sample	Raw Data Reads	Clean Data Reads	Clean Data Bases (bp)	Clean Data Rate (%)	Q20 rate (%)	Q30 rate (%)	GC Content (%)
Leaf 1 (L1, January)	21624453	21414736	6.43G	99.97	97.96	93.69	44.68
Leaf 2 (L2, February)	23174874	22954765	6.89G	99.97	97.82	93.37	44.88
Leaf 3 (L3,March)	22203308	21999586	6.60G	99.97	97.92	93.56	44.64
Leaf 5 (L5, May)	22024312	21850380	6.55G	99.97	97.68	93.03	45.18
Leaf 7 (L7, July)	22158667	21935984	6.58G	99.97	97.92	93.60	44.92
Leaf 10 (L10, October)	22691028	22465809	6.74G	99.97	97.78	93.26	44.85
Female cone 1 (F1, January)	23067315	22878178	6.86G	99.97	97.92	93.58	45.06
Female cone 2 (F2, February)	23228318	23006404	6.90G	99.97	97.79	93.28	44.87
Female cone 3 (F3, March)	21693451	21540598	6.46G	99.97	97.82	93.35	45.00
Female cone 5 (F5, May)	24509500	24336112	7.30G	99.97	97.75	93.16	44.90
Female cone 7 (F7, July)	21774984	21566613	6.47G	99.97	97.94	93.67	45.69
Female cone 10 (F10, October)	20955037	20772465	6.23G	99.97	97.81	93.32	45.22
**Average**	22425437.22	22226802.61	6.67G	99.97	97.84	93.41	44.99
