# Supplementary material for: Morpho-histology, endogenous hormone dynamics, and transcriptome profiling in Dacrydium pectinatum during female cone development
Source: Front Plant Sci. 2022 Aug 17;13:954788. doi: 10.3389/fpls.2022.954788 (PMC9428629; doi:10.3389/fpls.2022.954788)
Supplement: Supplementary file 9 [file Data_Sheet_9.PDF]

Supplementary Table 3. KEGG pathway annotation of *Dacrydium pectinatum* unigenes.

Pathway	Pathway ID	Number of unigenes	Percentage (%)
Ribosome	ko03010	5717	5.64
Carbon metabolism	ko01200	3277	3.24
Protein processing in endoplasmic reticulum	ko04141	2517	2.49
Biosynthesis of amino acids	ko01230	2286	2.26
Spliceosome	ko03040	2112	2.09
RNA transport	ko03013	1745	1.72
Oxidative phosphorylation	ko00190	1657	1.64
Endocytosis	ko04144	1446	1.43
Glycolysis / Gluconeogenesis	ko00010	1338	1.32
Glyoxylate and dicarboxylate metabolism	ko00630	1200	1.18
Citrate cycle (TCA cycle)	ko00020	1104	1.09
Pyruvate metabolism	ko00620	1078	1.06
Longevity regulating pathway - multiple species	ko04213	1020	1.01
Antigen processing and presentation	ko04612	1020	1.01
Purine metabolism	ko00230	1003	0.99
Estrogen signaling pathway	ko04915	993	0.98
Peroxisome	ko04146	980	0.97
Fatty acid metabolism	ko01212	960	0.95
PI3K-Akt signaling pathway	ko04151	929	0.92
Valine, leucine and isoleucine degradation	ko00280	880	0.87
