# Supplementary material for: Morpho-histology, endogenous hormone dynamics, and transcriptome profiling in Dacrydium pectinatum during female cone development
Source: Front Plant Sci. 2022 Aug 17;13:954788. doi: 10.3389/fpls.2022.954788 (PMC9428629; doi:10.3389/fpls.2022.954788)
Supplement: Supplementary file 10 [file Data_Sheet_10.PDF]

**Supplementary Table 4.** Differentially expressed unigenes during *D. pectinatum* Female cone development that are involved in plant hormone biosynthesis or metabolism pathway.

| Plant hormones | Gene ID | | Annotated genes |
|---|---|---|---|
| IAA | Cluster-104030.22157 | *AUX1* | *Auxin transporter-like protein 1* |
| | Cluster-104030.31842 | | |
| | Cluster-104030.16701 | | |
| | Cluster-104030.36507 | | |
| | Cluster-104030.18538 | | |
| | Cluster-115312.0 | *GH3* | *Indole-3-acetic acid-amido synthetase* |
| | Cluster-104030.26212 | *SAUR* | *Small auxin up-regulated genes* |
| | Cluster-104030.25927 | | |
| | Cluster-116754.0 | | |
| | Cluster-104030.20773 | | |
| | Cluster-120469.0 | | |
| | Cluster-104030.20959 | | |
| | Cluster-104030.25056 | | |
| | Cluster-104030.24156 | | |
| | Cluster-104030.30912 | | |
| | Cluster-104030.19057 | | |
| | Cluster-104030.8243 | | |
| | Cluster-104030.8984 | | |
| | Cluster-116953.0 | | |
| | Cluster-118975.0 | | |
| | Cluster-104030.7860 | | |
| | Cluster-104030.30598 | | |
| | Cluster-118079.0 | | |
| | Cluster-104030.7587 | | |
| | Cluster-104030.6249 | | |
| CTK | Cluster-98017.0 | *AHP* | *histidine-containing phosphotransfer factor* |
| | Cluster-114155.0 | | |
| GA | Cluster-104030.22536 | *GID1* | *Gibberellin-insensitive dwarf 1* |
| | Cluster-75764.0 | *GID2* | *Gibberellin-insensitive dwarf 2* |
| | Cluster-104030.17258 | | |
| | Cluster-104030.14077 | | |
| | Cluster-104030.29271 | *DELLA* | *GRAS family transcription factor* |
| | Cluster-104030.14265 | | |
| ABA | Cluster-104030.14178 | *PYR/PYL* | *regulatory component of ABA receptor 1* |

| | | | |
|---|---|---|---|
| | Cluster-104030.13081<br>Cluster-104030.31553<br>Cluster-104030.4273<br>Cluster-104030.35093<br>Cluster-104030.12023 | | |
| | Cluster-90765.1<br>Cluster-104030.23132<br>Cluster-104030.18273<br>Cluster-104030.21435<br>Cluster-104030.28174 | *PP2C* | *highly ABA-induced PP2C protein 2* |
| | Cluster-104030.23551 | *SNRK2* | *serine/threonine-protein kinase* |
| | Cluster-104030.22979 | *ABF* | *ABA responsive element binding factor* |
| Ethylene | Cluster-104030.7802<br>Cluster-104030.32717<br>Cluster-104030.36074 | *ETR* | *ethylene receptor* |
| | Cluster-67326.2<br>Cluster-104030.21931<br>Cluster-71378.1 | *MPK6* | *mitogen-activated protein kinase 6* |
| | Cluster-104030.25549 | *EBF1/2* | *EIN3-binding F-box 1/2* |
| | Cluster-104030.16464 | *ERF1* | *ethylene-responsive transcription factor 1* |
